# Potential Disease-Modifying Effects of Lithium Carbonate in Niemann-Pick Disease, Type C1

**DOI:** 10.3389/fphar.2021.667361

**Published:** 2021-06-09

**Authors:** Shiqian Han, Huiwen Zhang, Mengni Yi, Xiaoqing Liu, Gustavo H. B. Maegawa, Yunding Zou, Qijun Wang, Dianqing Wu, Zhijia Ye

**Affiliations:** ^1^ Department of Tropical Medicine, College of Preventive Medicine, Army Medical University (Third Military Medical University), Chongqing, China; ^2^ Department of Pediatric Endocrinology and Genetics, Xinhua Hospital, Shanghai Institute for Pediatric Research, Shanghai Jiao Tong University School of Medicine, Shanghai, China; ^3^ Department of Pediatrics, Genetics and Metabolism, University of Florida, Gainesville, FL, United States; ^4^ Department of Hematology, Southwest Hospital, Army Medical University (Third Military Medical University), Chongqing, China; ^5^ Department of Gastroenterology of Ruijin Hospital, Shanghai Institute of Immunology, Shanghai Jiao Tong University School of Medicine, Shanghai, China; ^6^ Departments of Vascular Biology and Therapeutics Program, Yale University School of Medicine, New Haven, CT, United States; ^7^ Department of Pharmacology, Yale University School of Medicine, New Haven, CT, United States; ^8^ Laboratory Animal Research Center, Chongqing University School of Medicine, Chongqing, China

**Keywords:** niemann-pick disease, lithium, clinical trial, 7-ketocholesterol, NNSS

## Abstract

**Background:** Niemann-Pick disease type C1 (NP-C1) is a rare, autosomal-recessive neurodegenerative disorder with no United States Food and Drug Administration (FDA)-approved drug. Lithium has been shown to have considerable neuroprotective effects for neurological disorders such as bipolar disorder, Alzheimer’s disease and stroke and has been tested in many clinical trials. However, the pharmacological effect of lithium on NP-C1 neurodegenerative processes has not been investigated. The aim of this study was to provide an initial evaluation of the safety and feasibility of lithium carbonate in patients with NP-C1.

**Methods:** A total of 13 patients diagnosed with NP-C1 who met the inclusion criteria received lithium orally at doses of 300, 600, 900, or 1,200 mg daily. The dose was reduced based on tolerance or safety observations. Plasma 7-ketocholesterol (7-KC), an emerging biomarker of NP-C1, was the primary endpoint. Secondary endpoints included NPC Neurological Severity Scores (NNSS) and safety.

**Results:** Of the 13 patients with NP-C1 (12–33 years) enrolled, three withdrew (discontinuation of follow-up outpatient visits). The last observed post-treatment values of 7-KC concentrations (128 ng/ml, SEM 20) were significantly lower than pretreatment baselines values (185 ng/ml, SEM 29; *p* = 0.001). The mean NNSS was improved after lithium treatment at 12 months (*p* = 0.005). Improvement in swallowing capacity was observed in treated patients (*p* = 0.014). No serious adverse events were recorded in the patients receiving lithium.

**Conclusion:** Lithium is a potential therapeutic option for NP-C1 patients. Larger randomized and double-blind clinical trials are needed to further support this finding.

**Clinical Trial Registration:** ClinicalTrials.gov, NCT03201627.

## Introduction

Niemann-Pick disease, type C1 (NP-C1) is a rare and progressive neurodegenerative lysosomal disease caused by mutations in *NPC1*. Cells with a defect in NPC1 lead to the endo/lysosomal accumulation of unesterified cholesterol and glycosphingolipids (Ory, 2000; Vanier, 2010). NP-C1 is a multisystemic inborn lysosomal disorder, and its clinical manifestations include neurological, visceral and psychiatric symptoms and signs (Vanier, 2010). The infantile-onset clinical form of NP-C1 is characterized by visceral symptoms and global neurodevelopmental delay, while late onset forms of NP-C1 with adolescent/adult-onset forms present with a wide spectrum of neurological and psychiatric manifestations (Bonnot et al., 2019). The neurological component of NP-C1 consists mostly of dysarthria, dysphagia, cerebellar ataxia and insidious dementia. The majority of affected patients typically present with vertical supranuclear gaze palsy (Solomon et al., 2005). This progressive neurodegeneration of NP-C1 coupled with delayed diagnosis indicates that manifestation improvement and stabilization and deterioration progression reduction are the priority attainable goals for long-term therapy (Rego et al., 2019).

Miglustat is the only available treatment for NP-C1 patients. Miglustat can stabilize neurological manifestations by inhibiting glycosphingolipid biosynthesis (Patterson et al., 2010; Wraith et al., 2010) and was approved in Europe, Canada, Japan and China but not in the United States. 2-Hydroxypropyl-β-cyclodextrins (HPβCD) has been shown to ameliorate neurological signs and prolong lifespan in both mouse (Liu et al., 2009) and cat (Vite et al., 2015) models of NP-C1. HPβCD is currently in phase II/III clinical trials. However, the adverse side effects, such as ototoxicity (Crumling et al., 2012; Vite et al., 2015), remain issues that need to be addressed. The emerging approaches for NP-C1 treatment, including arimoclomol (Kirkegaard et al., 2016), histone deacetylase inhibitor therapy (Kim et al., 2007; Alam et al., 2016) and gene therapy (Chandler et al., 2017; Xie et al., 2017; Hughes et al., 2018), have shown significant therapeutic potential in preclinical and clinical studies. Nevertheless, treatment options for NP-C1 are very limited, and new therapies or strategies are needed.

Over the last 2 decades, burgeoning evidence of beneficial neurotrophic and neuroprotective effects of lithium salt has emerged (Rybakowski et al., 2018). Lithium is the first-line mood stabilizer for the treatment of bipolar disorder (McIntyre et al., 2020). Lithium was also demonstrated to improve phenotypes in animal models of clinical neurodegenerative conditions, including Alzheimer’s disease (Fiorentini et al., 2010; Leroy et al., 2010; Zhang et al., 2011), Huntington’s disease (Wei et al., 2001; Senatorov et al., 2004), Parkinson’s disease (Youdim and Arraf, 2004; Qi et al., 2017), and stroke (Doeppner et al., 2017). Recently, hyperphosphorylated tau was observed in NP-C1 postmortem brains and in patient-derived neurons (Burbulla et al., 2021). Lithium, a selective inhibitor of glycogen synthase kinase 3β (GSK3β), could significantly decrease hyperphosphorylation of tau (Madra and Sturley, 2010). Moreover, the combination of bipolar medications, including lithium, was reported to increase the expression of NPC1 in a human neuronal cell line (Kidnapillai et al., 2019). The preceding study provided a promising way to investigate lithium as a treatment for NP-C1 patients. In this study, we conducted an open-label, nonrandomized and single-arm study to provide an initial evaluation of the safety and potential efficacy of lithium in patients with NP-C1.

## Methods

### Participants

A pilot open-label, nonrandomized, single-arm study was performed to assess the safety and clinical efficacy of oral lithium carbonate in patients with NP-C1 (ClinicalTrials.gov Identifier: NCT03201627) from July 2017 to November 2019. All patients were recruited at Xinhua Hospital, Shanghai Jiao Tong University School of Medicine, China. Patients with NP-C1 were strictly selected by the inclusion and exclusion criteria (Supplementary Table S1). Eligible patients with two mutant *NPC1* alleles had neurological manifestations, were aged 7 years or older, and were required to participate in all aspects of the clinical study. All patients or their legal guardians gave written informed consent prior to their participation in the study.

### Assignment and Procedures

Patients were sequentially assigned to receive a starting dose of 300 mg or 600 mg oral lithium carbonate pills with slow release. The lithium blood content was assessed one week after taking carbonate lithium for full clinical efficacy. Then patients had an outpatient visit once per month or every three months and received four phone calls every month during the treatment period. Any dose adjustment was made through a comprehensive decision combined with treatment efficacy and side effects. The NPC Neurological Severity Score (NNSS) were assessed at baseline and then approximately every three months or every 6 months. The NNSS is a Likert-like scale that assesses the neurological progression of NP-C1 in nine major and eight minor domains (Yanjanin et al., 2010; Ory et al., 2017).

### Outcomes

The primary outcome was the change of 7-Ketocholesterol (7-KC) concentration in plasma after drug administration compared with the 7-KC values before oral lithium carbonate administration. The quantification analysis of plasma 7-KC was carried out with LC/MS-MS by following the protocol we previously established (Lin et al., 2014; Zhang et al., 2014). The plasma lithium value was measured by IC-MS. Blood samples were obtained at fasting state before intake of morning medication (trough level).

The scoring of NNSS subdomains was performed based on the procedure recommended by the NIH (Ory et al., 2017). Two doctors who had more than 5 years of experience in NP-C1 diagnosis independently performed the NNSS assessments. The score was directly selected if the same score was given by two doctors, while the scoring was determined by discussion if different scores were given by two doctors.

The severity of adverse events was graded according to the Common Terminology Criteria for Adverse Events (CTCAE), Version 4.0.

### Statistical Analysis

The human study was a pre- and posttreatment design in which the baseline of a patient served as his/her own control. Patient characteristics were summarized using descriptive statistics. A paired *t*-test was used to assess changes in 7-KC concentrations. A nonparametric method, the Wilcoxon signed rank test, was used for the primary analysis for efficacy and the change in each subscale. Spearman correlation analysis was conducted to examine the association of some baseline characteristics and lithium concentration with changes in NNSS. Pearson’s correlation coefficient was used to assess the 7-KC data correlations.

Statistical analyses were performed with GraphPad Prism software (version 8.0.1). The statistical significance was set as *p* < 0.05, two-sided.

## Results

### Baseline Characteristics of the Patients

**FIGURE 1:**
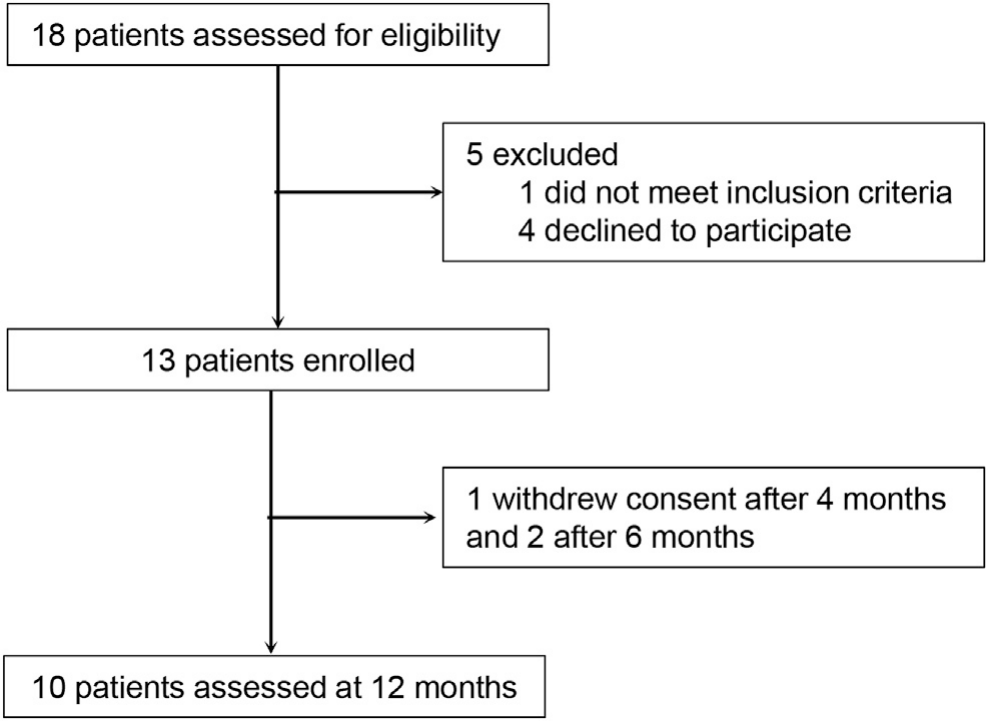
CONSORT flow diagram of patient recruitment until the 12-months follow-up.

A total of 18 patients with NP-C1 aged 12 years or older were recruited for this single-arm pilot clinical study to assess the safety and clinical efficacy of oral lithium carbonate (Figure 1). Five patients were excluded due to an inability to comply with the study procedures (four patients) or a failure to meet the inclusion criteria (one patient). 13 patients were eligible and were enrolled in the study. Two patients were followed up only for 6 months and one patient was able to complete the 4-month assessment due to the discontinuation of follow-up outpatient visits. The remaining 10 patients were followed up for 12 months. The baseline characteristics are provided in Table 1 and Supplementary Table S2. We used the last follow-up data as the endpoint for these 13 patients who took lithium carbonate.

**TABLE 1:** Baseline characteristics of patients.

Characteristic	Total patients (*n* = 13)
**Age at baseline (years)**	
Mean (SD)	19 (6.65)
Range	12–33
**Sex**	
Male (%)	7 (54%)
Female (%)	6 (46%)
**Age of first neurological symptom, years**	
Mean (SD)	9.62 (2.47)
Range	7–13
**Age of diagnosis, years**	
Mean (SD)	18.15 (6.57)
Range	10–31
**Miglustat use, n (%)**	
Yes	2 (15%)
No	11 (85%)

**FIGURE 2:**
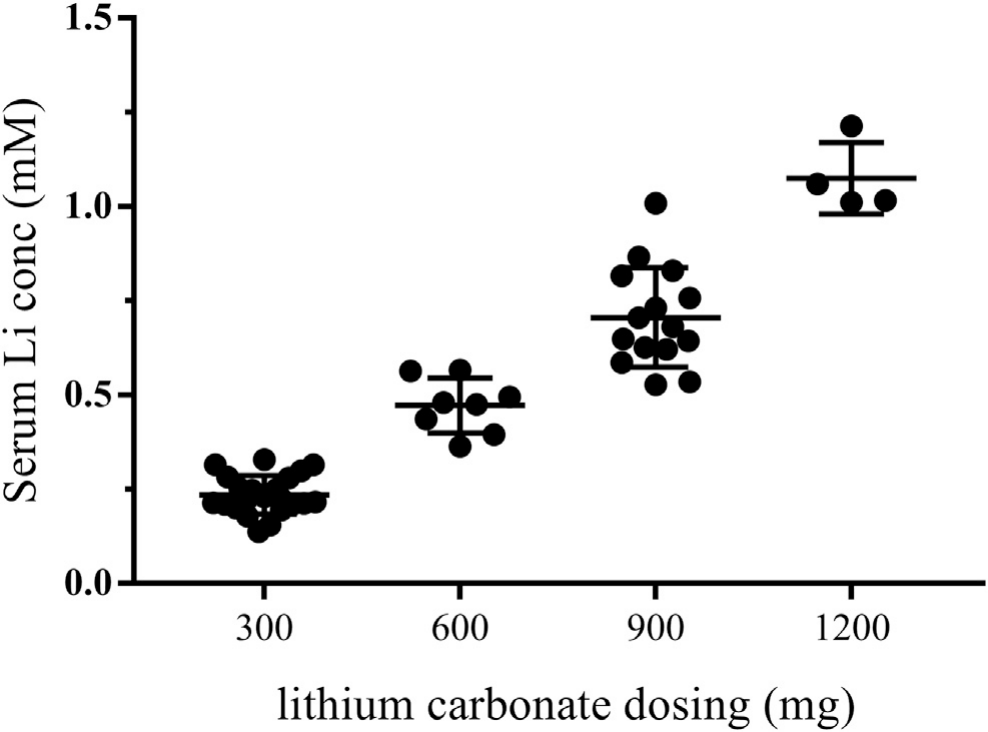
Plasma lithium levels after receiving lithium carbonate. Every point represents that plasma lithium levels were detected after receiving lithium at doses of 300, 600, 900, or 1,200 mg. The first month was the dose adjustment period. and plasma lithium levels were measured by IC-MS once a week. The plasma lithium levels were then examined approximately every 3 months after the first month. Therefore, the lithium plasma levels of one patient were detected several times at one dose. conc, concentration.

During the first month of the treatment (the dose adjustment period), the patients were given lithium doses ranging from 300 to 900 mg/day, and serum lithium levels were measured at least once a week. Detailed lithium dosing information is provided in Supplementary Table S3. The plasma lithium levels were then examined approximately every 3 months after the first month, and the participant’s plasma lithium concentrations were positively associated with oral doses of lithium carbonate (Figure 2). Because the initial goal was to reach the upper end of the therapeutic lithium concentrations range, i.e., between 0.6 and 1.0 mM, which is expected under treatment of bipolar disorder (Hopkins and Gelenberg, 2000), some patients received doses as high as 1,200 mg/day. The reduction of the lithium dose was guided by safety and tolerance observations.

### Reduction in 7-KC

**FIGURE 3:**
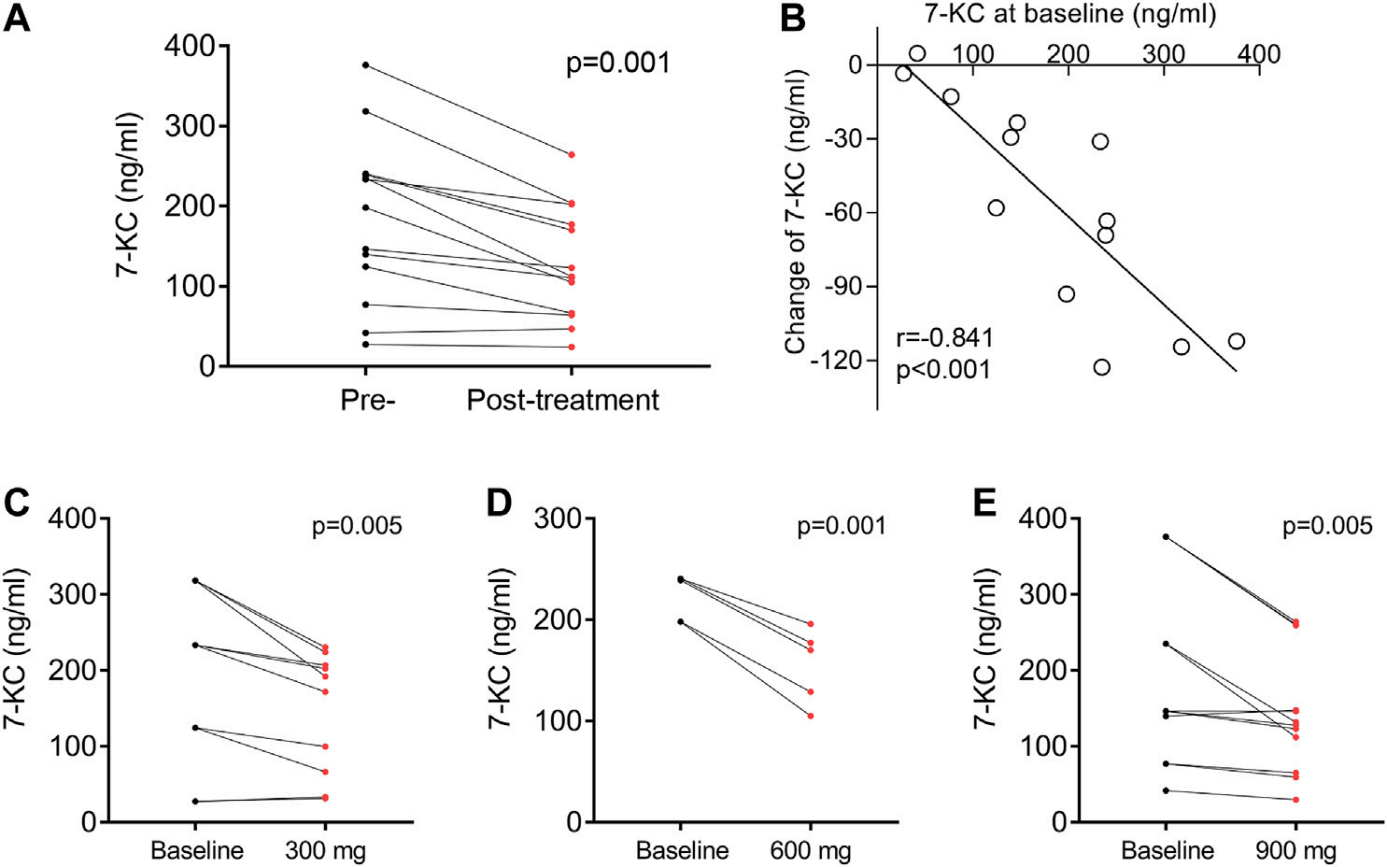
Effect of lithium on 7-KC. **(A)** Plasma 7-KC concentration measured before and at the last follow-up after lithium administration in 13 patients. **(B)** The association of plasma 7-KC at baseline with changes in 7-KC. Concentration change of plasma 7-KC after oral doses of 300 mg **(C)**, 600 mg **(D)** or 900 mg **(E)** of lithium carbonate. Each paired set of pre- and post-values represent an individual dose: 10 doses among four patients for 300 mg, five doses among three patients for 600 mg and 12 doses among six patients for 900 mg r is the Pearson correlation coefficient.

In our previous research, we reported that the cholesterol oxidation product 7-KC was detected at elevated levels in NP-C1 patients (Lin et al., 2014; Zhang et al., 2014), and that 7-KC could serve as a specific biomarker for the disease (Porter et al., 2010; Stampfer et al., 2013). This study included measurements of plasma 7-KC concentrations before and after lithium study administration. The last observed posttreatment values of 7-KC concentrations (128 ng/ml, SEM 20) were significantly decreased compared with the pretreatment baseline values (185 ng/ml, SEM 29; *p* = 0.001) (Figure 3A). Importantly, higher baseline 7-KC concentrations were significantly correlated with greater reductions in the 7-KC concentration (Figure 3B; *r* = −0.841; *p* < 0.001). We also found that 7-KC significantly decreased after oral administration of 300, 600 and 900 mg lithium carbonate tablets (Figures 3C–E), but plasma lithium concentration was not significantly correlated with the change in 7-KC concentration (Supplementary Figure S1, *r* = 0.028; *p* = 0.928).

### NPC Neurological Severity Score

**FIGURE 4:**
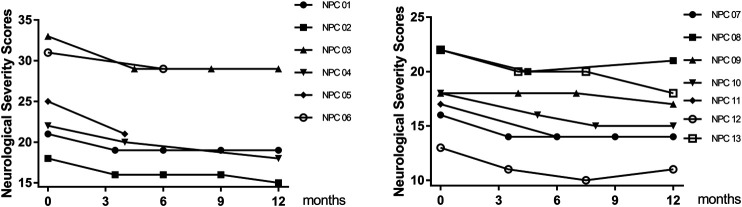
Effect of lithium on NNSS. The total NNSS of 13 patients who accepted lithium treatment obtained at multiple time points within 12 months.

**FIGURE 5:**
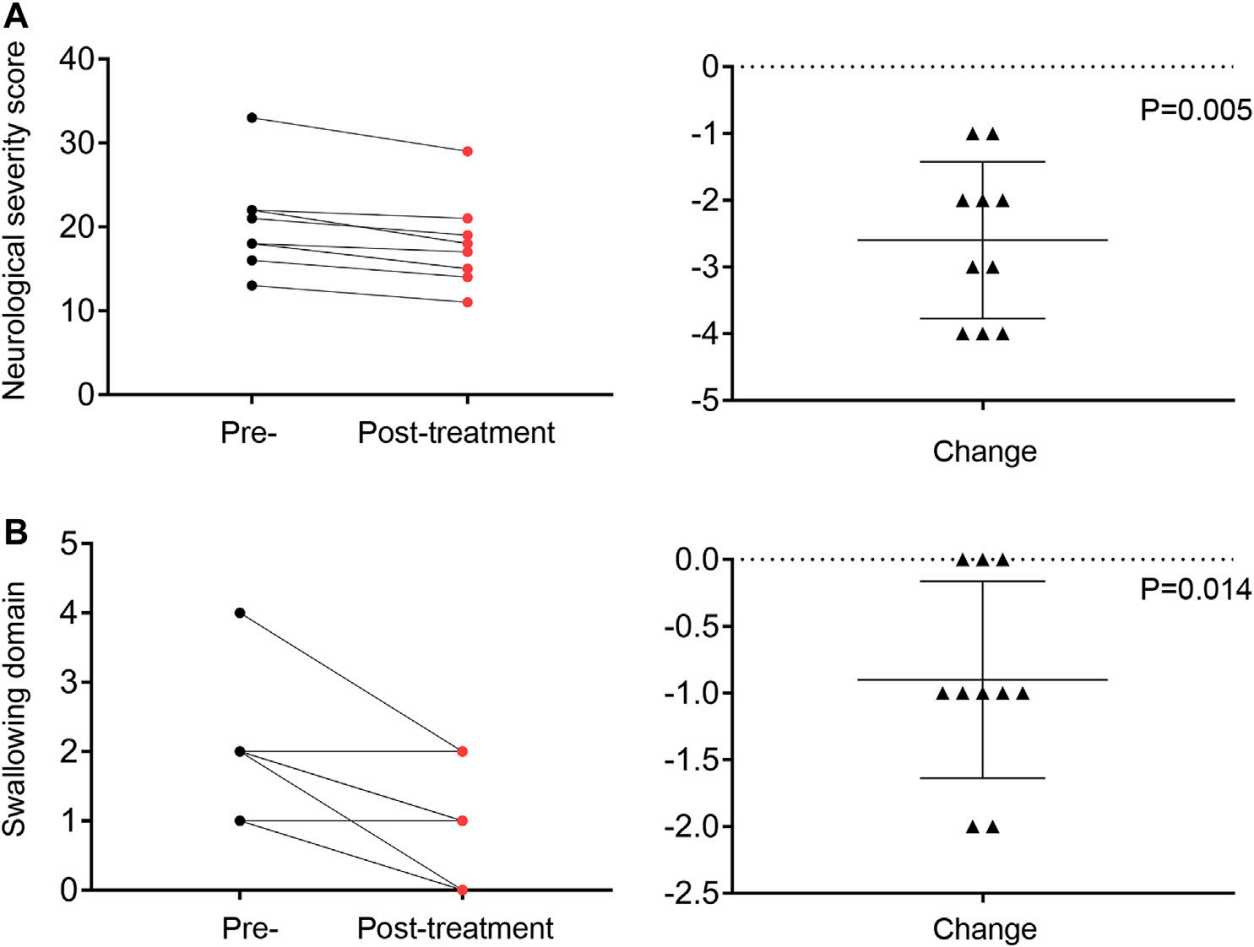
The total NNSS **(A)** and swallowing **(B)** domain score recorded at the pre-study and 12-months visits. Change in total score and swallowing domain score from the pre-study to the final visit assessment.

Clinical benefit was ascertained by comparing the total NNSS before lithium treatment with that after lithium treatment. Ten patients were assessed at 12 months. All 10 patients achieved reductions in their NNSS after 12-months, with an average 2.6-point decrease (*p* = 0.005) (Table 2). Among the 17 NNSS domains examined, the reduction in the swallowing domain score was statistically significant (*p* = 0.014). There were trends of reduction in incontinence scores (*p* = 0.083) (Table 2). The examination of the NNSS at the interim time points in these 13 lithium-treated patients revealed no substantial deleterious effect of lithium (Figure 4, Supplementary Table S4). The decrease in the total NNSS was mainly contributed by improvement in the swallowing domain score, assessing the level of dysphagia (Figures 5A,B). Natural history studies have shown that swallowing ability is gradually impaired from the time of disease onset (Wraith et al., 2009). Together, these results suggest that lithium may be an available therapeutic option for NP-C1 patients. Other characteristics, including sex, age at diagnosis, the age of onset, length of treatment duration (data not shown), and lithium concentration, failed to significantly correlate with the reduction of scores in the NNSS (Supplementary Figure S2, *r* = −0.219; *p* = 0.472).

**TABLE 2:** Comparison of NNSS subscales before and after 12 months of lithium treatment in 10 NP-C1 patients.

	Prior treatment (SD)	After treatment (SD)	*p* value
**Eye movement**	2.7 (0.5)	2.7 (0.5)	-
**Ambulation**	2.2 (0.6)	2.0 (0)	0.317
**Speech**	1.4 (0.7)	1.3 (0.7)	0.317
**Swallowing**	1.8 (0.9)	0.9 (0.7)	**0.014**
**Fine motor**	2.1 (0.7)	2.0 (0.8)	0.317
**Cognition**	3.6 (1.0)	3.4 (1.0)	0.157
**Hearing**	0.7 (1.3)	0.6 (1.1)	0.317
**Memory**	1.7 (0.8)	1.7 (0.8)	-
**Seizures**	0.6 (1.3)	0.5 (1.1)	0.317
**Gelastic cataplexy**	0.4 (0.8)	0.2 (0.4)	0.157
**Hyperreflexia**	1.2 (0.6)	1.1 (0.7)	0.317
**Narcolepsy**	0 (0)	0 (0)	-
**Incontinence**	0.6 (0.7)	0.3 (0.7)	0.083
**Behavior**	0 (0)	0 (0)	-
**Auditory brain response (ABR)**	1.0 (0)	1.0 (0)	-
**Psychiatric**	0.3 (0.7)	0 (0)	0.18
**Respiratory**	0 (0)	0 (0)	-
**Total score**	20.3 (5.4)	17.7 (4.9)	**0.005**

The bold values mean p<0.05.

### Safety Evaluation

Lithium treatment was generally well tolerated in NP-C1 patients. Adverse events, including hypothyroidism (3 patients) and benign leukocytosis (5 patients), as well as rare occurrences of manic episodes (2 patients) and drooling (1 patient), were observed at high doses (Table 3).

**TABLE 3:** Selected adverse events.

Event-no. (%)	Subjects (*n* = 13)
Mania episode	2 (15.4)
Increased drooling	1 (7.7)
Benign leukocytosis	5 (38.5)
Transient hypothyroidism	3 (23.1)

## Discussion

Lithium is an agent that has been in use for the treatment of psychiatric disorders for decades after research by John Cade, an Australian psychiatrist (Cade, 1949). To our knowledge, this is the first open label study to assess the safety and potential efficacy of lithium treatment in patients with NP-C1. Our results preliminarily demonstrated that lithium carbonate tablets may have the potential to ameliorate the neurological phenotypes of NP-C1 patients. Lithium treatment significantly improved overall NNSS scores and swallowing subdomain scores. Furthermore, lithium significantly reduced plasma 7-KC in patients with NP-C1. We also demonstrated that lithium was well tolerated in patients with NP-C1. The lower incidence of side effects in this study was due to the low doses of lithium used in the NP-C1 patients.

Dysphagia was present in 55–80% of NP-C1 patients and was identified as one of the greatest risk factors for NP-C1 (Walterfang et al., 2012). Dysphagia is a main cause of aspiration pneumonia, contributing to two-thirds of deaths in NP-C1 patients (Petroianni et al., 2006; Walterfang et al., 2012). The improvement in swallowing capacity was closely related to the quality of life of NP-C1 patients. A recent study demonstrated that the determination of swallowing capacity could be considered as a clinically functional outcome indicator for future therapeutic interventions in NP-C1 (Solomon et al., 2020). In our study, most NP-C1 patients suffered from dysphagia during the disease course. Lithium carbonate significantly reduced the swallowing subdomain scores, indicating that lithium treatment may improve swallowing capacity and quality of life in NP-C1 patients. Cerebellar ataxia is one of the most specific manifestations in NP-C1 patients, and it correlates with gait ataxia, dysarthria, dysphagia and swallowing (Schmahmann, 2004). In preliminary studies, we demonstrated that lithium carbonate treatment significantly protected cerebellar Purkinje cells, improved food intake and extended survival *in vivo* studies using *NP-C1* ^*−/−*^ and *NP-C1* ^*I1061T*^ mouse models (unpublished data). Therefore, we speculate that lithium may improve swallowing difficulties by protecting Purkinje cell in the NP-C1 patients. Moreover, in a 48-weeks phase 2 trial of the efficacy of lithium in patients with spinocerebellar ataxia type 3 (an adult-onset disorder featured by cerebellar ataxia), ataxic manifestations at endpoint were significantly delayed in the treated group compared to the placebo group, and it was suggested that lithium could be beneficial to patients at a relatively early stage of the disease and to specific symptoms, for example cerebellar dysfunction (Saute et al., 2014).

Our biomarker data also support the clinical efficacy of lithium. The plasma content of oxysterol chemicals, including 7-KC, has been shown to be highly sensitive and specific in the detection of NP-C1 (Lin et al., 2014; Zhang et al., 2014). The plasma levels of 7-KC have significant correlations with different levels of disease severity of NP-C1 patients (Porter et al., 2010; Stampfer et al., 2013), and the decrease of 7-KC was beneficial to NPC1 mutant feline model after treated by HPβCD (Porter et al., 2010). Our data showed that plasma 7-KC was significantly reduced after oral doses of 300, 600 and 900 mg of lithium. In addition, higher baseline levels of plasma 7-KC were significantly correlated with greater reductions in patients after lithium administration. These findings raise the possibility that lithium may ameliorate the clinical NP-C1 severity. The mechanism underlying the effect of lithium on 7-KC is not clear. Lithium has been shown to attenuate DNA damage, mitochondrial dysfunction and lipid peroxidation by inhibiting GSK-3β and activating NRF-2 antioxidant response (Khairova et al., 2012; Castillo-Quan et al., 2016; Machado-Vieira, 2018). Lithium may lower 7-KC by modulating oxidative stress and lipid metabolism.

The interpretation of the changes in the NNSS results is limited in the context of a pilot study without placebo control. Considering the small sample size of this study, these potential effects of lithium definitely need more investigation in a larger randomized study. From a safety standpoint, no substantial deleterious effect of lithium was observed, including any adverse effect on motor and hearing features. Moreover, compared with other treatments for NP-C1, the advantages of lithium are readily available and relatively inexpensive.

We also explored oral doses of lithium between 300 and 1,200 mg per month and found no significant impact of average plasma lithium levels on the change in 7-KC levels and NNSS (Supplementary Figures S1, S2). A possible reason for this lack of correlation may be that the lithium dose even at 300 mg/day reached the plateau of the dose-response range. In future large studies, 300 mg/day may be considered the starting point.

In conclusion, we carried out an open-label pilot study to assess the safety, feasibility and tolerability of lithium carbonate in NP-C1 patients over a 12-month period. Lithium carbonate was safe and well tolerated and showed potential therapeutic effects on plasma 7-KC level reductions and swallowing improvement in NP-C1 patients. Notably, combination therapies of lithium with miglustat or HPβCD may have more potent therapeutic effects. Future prospective, randomized clinical trials with large sample sizes are needed to confirm this finding.

## Data Availability

The original contributions presented in the study are included in the article/Supplementary Material, further inquiries can be directed to the corresponding authors.
