# Supplementary material for: Potential Disease-Modifying Effects of Lithium Carbonate in Niemann-Pick Disease, Type C1
Source: Front Pharmacol. 2021 Jun 9;12:667361. doi: 10.3389/fphar.2021.667361 (PMC8220070; doi:10.3389/fphar.2021.667361)
Supplement: Supplementary file 1 [file DataSheet1.PDF]

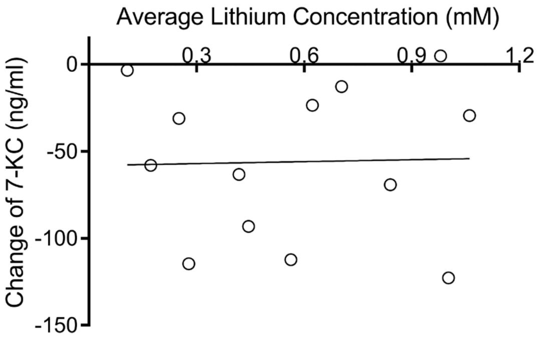

Figure S1. The association of lithium concentration with the change in 7-KC from the pre-study to the final visit assessment (Pearson r=0.028, p=0.928).

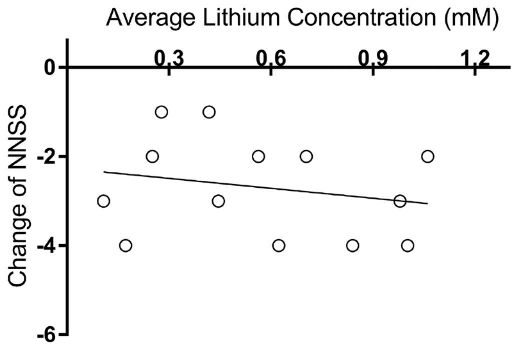

Figure S2. The association of lithium concentration with the change in NNSS from the pre-study to the final visit assessment (Pearson r=-0.219, p=0.472).

The inclusion criteria:
1. Age ≥7 and ≤ 40 years of age;
2. Two mutant NPC1 alleles;
3. Walk independently or need assistance to walk;
4. Subject(s) or guardian(s) must have the capacity to communicate with researchers, Understand and comply with study procedure;
5. The guardian can accompany the subject to participate in the study;
6. Written informed consent was obtained from each subject or guardian.
**The exclusion criteria:**
1. An individual neurological severity score ≥ 5;
2. Pregnant or breastfeeding;
3. Life expectancy ≤ 1 year;
4. Severe liver malfunction or renal failure;
5. No neurological symptoms;
6. Received antidiuretic hormone.

Supplementary Table 1. Inclusion and exclusion criteria of NP-C1 patients.

Patient	Age at baseline, years	Sex	Age of first neurological symptom (years)	Age of diagnosis (years)	Miglustat use	Amino acid change (allele 1)	Nucleotide change (allele 1)	Amino acid change (allele 2)	Nucleotide change (allele 2)
**NPC 01**	12	Female	7	10	No	p.S940L	c.2819C>T	p.G992W	c.2974G>T
**NPC 02**	33	Male	13	31	No	p.L335fx	c.1004delT	p.V1078L	c.3232G>T
**NPC 03**	15	Female	8	14	No	p.L783S	c.2348T>C	p.R404fs	c.1210delC
**NPC 04**	21	Male	10	20	No	p.W544Gfs*19	c.1630delTA	p.R607Q	c.1820G>A
**NPC 05**	16	Male	8	16	No	p.N1156S	c.3467A>G	p.A1054T	c.3160G>A
**NPC 06**	18	Female	10	17	No	p.G1015R	c.3043G>A	p.L1247S	c.3740G>A
**NPC 07**	15	Male	7	15	No	p.D771E	c.2313C>G		c.3591+1G>A
**NPC 08**	12	Female	7	12	No		2912-6C>A	p.G992W	c.2974G>T
**NPC 09**	31	Male	13	31	Yes	p.R161W	c.481C>T	p.R1077L	3230G>T
**NPC 10**	20	Male	12	19	No	p.S734G	c.2200A>G	p.R789H	c.2366G>A
**NPC 11**	22	Female	7	21	Yes	p.V768A	c.2303T>C	p.916del	c.2746-2748del
**NPC 12**	13	Female	10	12	No	p.G913D	c.2738G>A	p.D501Y	c.1501G>T
**NPC 13**	19	Male	13	18	No	p.D721V	c.2162A>T	p.D501Y	c.1501G>T

Supplementary Table 2. NP-C1 patients' demographics and clinical characteristics.

Subject	Month 1[a]	Month 2	Month 3	Month 4	Month 5	Month 6	Month 7	Month 8	Month 9	Month 10	Month 11	Month 12
**NPC 01**	300-600-900mg	900mg	900mg	900mg	1200mg	1200mg	1200mg	1200mg	1200mg	1200mg	1200mg	1200mg
**NPC 02**	600-900mg	900mg	900mg	900mg	1200mg	1200mg	1200mg	1200mg	1200mg	1200mg	1200mg	1200mg
**NPC 03**	300-600-900mg	900mg	900mg	900mg	900mg	900mg	900mg	900mg	900mg	900mg	900mg	900mg
**NPC 04**	600-900mg	900mg	900mg	900mg	900mg	900mg	900mg	900mg	900mg	900mg	900mg	900mg
**NPC 05**	300-600-900mg	900mg	900mg	600mg								
**NPC 06**	300-600-900mg	900mg	900mg	900mg	900mg	900mg						
**NPC 07**	300-600-900mg	900mg	900mg	900mg	900mg	900mg	900mg	900mg	900mg	900mg	900mg	900mg
**NPC 08**	300-600-900mg	900mg	900mg	900mg	600mg	600mg	600mg	600mg	600mg	600mg	600mg	600mg
**NPC 09**	300-600-900mg	900mg	900mg	300mg	300mg	300mg	300mg	300mg	300mg	300mg	300mg	300mg
**NPC 10**	300-600-300mg	300mg	300mg	300mg	300mg	300mg	300mg	300mg	300mg	300mg	300mg	300mg
**NPC 11**	300-600mg	600mg	600mg	600mg	600mg	600mg						
**NPC 12**	300mg	300mg	300mg	300mg	300mg	300mg	300mg	300mg	300mg	300mg	300mg	300mg
**NPC 13**	300mg	300mg	300mg	300mg	300mg	300mg	300mg	300mg	300mg	300mg	300mg	300mg

Supplementary Table 3. Lithium carbonate dosing of patients. a. The first month was the dose adjustment period.

	Total Score	Eye movement	Ambulation	Speech	Swallowing	Fine motor	Cognition	Hearing	Memory	Seizures	Gelastic Cataplexy	Hyperreflexia	Narcolepsy	Incontinence	Behavior	Auditory Brain Response (ABR)	Psychiatric	Respiratory
Neurological Severity Scores before treatment																		
**NPC 01**	21	3	2	1	1	2	4	0	2	0	2	2	0	0	0	1	1	0
**NPC 02**	18	3	2	1	2	2	4	0	1	0	0	1	0	1	0	1	0	0
**NPC 03**	33	3	4	3	4	4	4	0	3	3	0	2	0	2	0	1	0	0
**NPC 04**	22	3	2	2	2	2	4	0	2	0	0	2	0	0	0	1	2	0
**NPC 05**	25	2	2	2	4	4	4	0	2	0	0	2	0	1	0	1	0	1
**NPC 06**	31	3	2	3	2	4	4	0	4	0	0	2	2	2	0	1	0	2
**NPC 07**	16	2	2	1	1	2	4	0	2	0	0	1	0	0	0	1	0	0
**NPC 08**	22	3	2	2	2	2	4	0	3	0	2	1	0	0	0	1	0	0
**NPC 09**	18	2	2	1	1	2	4	3	1	0	0	1	0	0	0	1	0	0
**NPC 10**	18	3	2	1	1	2	4	1	1	0	0	1	0	1	0	1	0	0
**NPC 11**	17	2	1	1	1	1	1	0	2	0	0	1	0	0	1	1	2	0
**NPC 12**	13	2	2	1	2	1	1	0	1	0	0	1	0	1	0	1	0	0
**NPC 13**	22	3	2	1	2	2	3	3	1	3	0	0	0	1	0	1	0	0
Neurological Severity Scores after treatment																		
**NPC 01**	19	3	2	1	1	2	4	0	2	0	1	2	0	0	0	1	0	0
**NPC 02**	15	3	2	1	0	2	4	0	1	0	0	1	0	0	0	1	0	0
**NPC 03**	29	3	2	3	2	4	4	0	3	3	0	2	0	2	0	1	0	0
**NPC 04**	18	3	2	1	1	2	4	0	2	0	0	2	0	0	0	1	0	0
**NPC 05**	21	2	2	2	1	4	4	0	2	0	0	2	0	0	0	1	0	1
**NPC 06**	29	3	2	3	1	4	3	0	4	0	0	2	2	2	0	1	0	2
**NPC 07**	14	2	2	1	0	1	4	0	2	0	0	1	0	0	0	1	0	0
**NPC 08**	21	3	2	2	2	2	4	0	3	0	1	1	0	0	0	1	0	0
**NPC 09**	17	2	2	1	1	2	3	3	1	0	0	1	0	0	0	1	0	0
**NPC 10**	15	3	2	1	0	2	3	1	1	0	0	0	0	1	0	1	0	0
**NPC 11**	14	2	1	1	1	1	1	0	2	0	0	1	0	0	0	1	0	0
**NPC 12**	11	2	2	1	1	1	1	0	1	0	0	1	0	0	0	1	0	0
**NPC 13**	18	3	2	1	1	2	3	2	1	2	0	0	0	0	0	1	0	0

Supplementary Table 4. Neurological severity scores at baseline and 12-month or last follow-up after lithium treatment.
